# Reproductive factors as risk modifiers of breast cancer in *BRCA* mutation carriers and high-risk non-carriers

**DOI:** 10.18632/oncotarget.22193

**Published:** 2017-10-31

**Authors:** Boyoung Park, John L. Hopper, Aung K. Win, James G. Dowty, Ho Kyung Sung, Choonghyun Ahn, Sung-Won Kim, Min Hyuk Lee, Jihyoun Lee, Jong Won Lee, Eunyoung Kang, Jong-Han Yu, Ku Sang Kim, Byung-In Moon, Wonshik Han, Dong-Young Noh, Sue K. Park, KOHBRA Study Group

**Affiliations:** ^1^ Department of Cancer Control and Population Health, National Cancer Center Graduate School of Cancer Science and Policy, Gyeonggi-Do, Korea; ^2^ National Cancer Control Institute, National Cancer Center, Gyeonggi-Do, Korea; ^3^ Centre for Epidemiology and Biostatistics, Melbourne School of Population and Global Health, The University of Melbourne, Parkville, Victoria, Australia; ^4^ Department of Preventive Medicine, Seoul National University College of Medicine, Seoul, Korea; ^5^ Cancer Research Institute, Seoul National University, Seoul, Korea; ^6^ Department of Biomedical Science, Seoul National University Graduate School, Seoul, Korea; ^7^ Department of Surgery, Daerim-Sungmo Hospital, Seoul, Korea; ^8^ Department of Surgery, College of Medicine, Soonchunhyang University, Seoul, Korea; ^9^ Department of Surgery, College of Medicine, University of Ulsan and Asan Medical Center, Seoul, Korea; ^10^ Department of Surgery, Breast and Endocrine Service, Seoul National University Bundang Hospital, Gyeonggi-Do, Korea; ^11^ Division of Breast and Endocrine Surgery, Department of Surgery, Samsung Medical Center, Sungkyunkwan University School of Medicine, Seoul, Korea; ^12^ Breast-Thyroid Center, Ulsan City Hospital, Ulsan City Hospital Group, Ulsan, Korea; ^13^ Department of Surgery, Ewha Womans University Hospital, Seoul, Korea; ^14^ Department of Surgery, Seoul National University College of Medicine, Seoul, Korea

**Keywords:** breast neoplasm, reproductive factors, BRCA1/2 mutation carriers, familial breast cancer, early onset breast cancer

## Abstract

This study was conducted to identify the role of reproductive factors as environmental modifiers for breast cancer (BC) risk in clinic-based, East-Asian *BRCA1* and *BRCA2* mutation carriers and non-carriers with high-risk criteria of *BRCA* mutations (family history (FH) of BC, early-onset BC (aged ≤40 years)). A total of 581 women who were *BRCA* carriers (222 *BRCA1* and 359 *BRCA2*), 1,083 non-carriers with FH, and 886 non-carriers with early-onset BC were enrolled and interviewed to examine the reproductive factors, from 2007 to 2014. The hazard ratio (HR) and its 95% confidence interval (CI) in the weighted Cox regression model were used to calculate the BC risk based on the reproductive factors. Earlier menarche increased BC risk by 3.49-fold in *BRCA2* mutation carriers (95%CI=2.03–6.00) and 3.30-fold in non-carriers with FH (95%CI=1.73–6.34), but was insignificantly associated with *BRCA1* carriers and non-carriers for early-onset BC (P-heterogeneity=0.047). Higher parity decreased BC risk in *BRCA* carriers and non-carriers with FH, especially in *BRCA1* carriers (HR=0.27, 95% CI=0.09–0.83 for two parity; and HR=0.23, 95%CI=0.05–1.00 for ≥3 parity), but increased the early-onset BC risk (HR=4.63, 95%CI=2.56–8.51 for >3 parity, p-heterogeneity=0.045). Oral contraceptive (OC) use and longer estrogen exposure periods (≥30 years) were associated with an increased risk of early-onset BC (HR=3.99, 95%CI=1.65–9.67; HR=7.69, 95%CI=1.96–25.01), while OC use was not associated with BC risk in other groups and longer estrogen exposure had rather decreased risk for BC risk (both p-heterogeneity<0.001). Several reproductive factors as risk modifiers could heterogeneously be associated with BC among *BRCA1/2* mutation carriers, non-carriers with FH, and early-onset BC non-carriers.

## INTRODUCTION

Germline mutations in the *BRCA1* or *BRCA2* genes are responsible for about 5% of breast cancer (BC) and are associated with a substantially increased lifetime risk of BC to 70 years old with approximately 65% and 45% of risk, respectively, in Caucasian populations [1, 2].

Reproductive factors, including lower number of parity, late parity, early age at menarche, and late menopausal age, are well-established risk factors of female BC in the general population [3, 4]. However, whether reproductive factors in the general population would act as risk factors for BC in *BRCA1/2* mutation carriers remain questionable, because *BRCA1/2* mutation can disrupt the estrogenic response in tissues by mutation itself [5] or an interaction with many other genes [6, 7]. Previous studies of BC risk based on the reproductive factors in *BRCA1/2* mutation carriers have produced inconsistent results; hence, the question remains [8–17]. Thus, the direction in the association of reproductive factors on BC risk in the general population has been hypothesized to be somewhat different from that in mutation carriers and genetically high-risk groups, such as familial BC or early-onset BC patients.

In particular, Asians have different BC-related characteristics from the Westerners. For example, Asians have a different distribution of genetic and environmental risk factors, such as lower incidence of BC and mortality rates, different age-specific incidence rate, poor prediction of BC assessment models developed in the Western populations, and higher prevalence of *BRCA2* than *BRCA1* mutations [18–20]. Therefore, identifying whether the effects of reproductive factors as risk modifiers of BC in *BRCA* mutation carriers are similar or not is necessary, regardless of ethnic differences. To date, few studies have focused on the effects of reproductive factors on BC for *BRCA1/2* mutation carriers in East-Asian population. The effect of reproductive factors on BC risk in the general population may be also different from that in genetically high-risk groups, such as familial BC or early-onset BC; however, previous studies on BC with family history (FH) or early-onset BC did not exist.

Thus, this study aimed to investigate the role of reproductive factors as risk modifiers of BC in *BRCA1/2* mutation carriers and hereditary high-risk groups without *BRCA1/2* mutations, such as non-carriers with FH of BC and non-carriers with early-onset BC in an East-Asian population.

## RESULTS

Table 1 shows the characteristics of female participants included in this study among the Korean Hereditary BC (KOHBRA) study. The BC patients with *BRCA2* mutation, non-carriers with FH of BC, and non-carriers with early-onset BC were older than the controls. The proportion of postmenopausal women was higher in *BRCA2* carrier BC patients than *BRCA2* carrier controls (*P* <0.05). In all groups, the proportion of current drinkers was lower in BC patients than controls (*P* < 0.05).

**Table 1:** Characteristics of female study participants with *BRCA 1/2* mutation carriers, non-carriers with family history of breast cancer, and non-carriers with early-onset breast cancer

	*BRCA1* mutation carriers		*BRCA2* mutation carriers		Non-carriers with family history of breast cancer		Non-carriers with early-onset breast cancer	
	Breast cancer	Control	Breast cancer	Control	Breast cancer	Control	Breast cancer	Control
	**Mean (SD)**	**Mean (SD)**	**Mean (SD)**	**Mean (SD)**	**Mean (SD)**	**Mean (SD)**	**Mean (SD)**	**Mean (SD)**
Age at participation^1^	40.2 (8.1)	39.5 (13.7)	46.3 (11.1)^2^	38.8 (13.1)^2^	46.2 (9.8)^2^	41.7 (14.1)^2^	35.2 (5.4)^2^	29.7 (5.8)^2^
	**N (%)**	**N (%)**	**N (%)**	**N (%)**	**N (%)**	**N (%)**	**N (%)**	**N (%)**
Postmenopausal women	40 (23.8)	13 (24.1)	112 (44.8)^2^	19 (17.4)^2^	303 (35.4)	46 (25.6)	54 (7.0)	1 (1.1)
Current drinkers	32 (19.1)^2^	17 (31.5)^2^	42 (16.8)^2^	45 (41.3)^2^	132 (15.4)^2^	87 (48.3)^2^	157 (20.3)^2^	56 (63.6)^2^
Current smokers	7 (4.2)	5 (9.3)	4 (1.6)	2 (1.8)	26 (3.0)	6 (3.3)	17 (2.2)	6 (6.8)
BMI ≥ 25 Kg/m^2^	28 (16.8)	8 (14.8)	46 (18.4)	23 (21.1)	172 (20.1)	32 (17.8)	108 (14.0)	9(10.2)

BMI body mass index, BC breast cancer, OC ovarian cancer, SD standard deviation, N number

^1^Age at diagnosis in breast cancer patients; Age at enrollment in controls

^2^p<0.05

Tables 2 and 3 show the associations between reproductive factors and BC risk in *BRCA1/2* mutation carriers, non-carriers with FH of BC, and non-carriers with early-onset BC. Increased number of parity was significantly associated with reduced risk of BC in *BRCA 1* mutation carriers (hazard ratio (HR)=0.27, 95% confidence interval (CI)=0.09–0.83 for two parity; HR=0.23, 95% CI=0.05–1.00 for ≥3 parity; p-trend <0.001) and increased risk for the early-onset BC in non-carriers (HR=4.63, 95% CI=2.56–8.51 for ≥3 parity). The associations among the four groups were statistically heterogeneous (P-heterogeneity <0.001). For women ≤40 years old, later age at first full-term pregnancy (FFTP) decreased the BC risk in *BRCA1* mutation carriers (HR=0.33, 95% CI=0.12–0.90 for 24–29 years old at FFTP; HR=0.14, 95% CI=0.03–0.66 for ≥30 years old at FFTP, compared with women aged ≤23 years at FFTP; p-trend <0.001). However, for women >40 years old, with FFTP between 24 and 29 years old, increased BC risk was observed in *BRCA2* mutation carriers compared with *BRCA2* mutation carriers whose age at FFTP was ≤23 years old (HR=3.24, 95% CI=1.43–7.40). In addition, a significant trend between later age at FFTP and BC risk in BRCA1 mutation carriers was also observed (p-trend =0.01). Oral contraceptive (OC) use had a 4.29-fold higher risk for early-onset BC in non-carriers (95% CI, 2.17–9.34), but was not associated with BC risk in other groups (P-heterogeneity among four groups = 0.045).

**Table 2:** Number of parity, age at first full-term pregnancy, and oral contraceptive use for the risk of breast cancer in *BRCA 1/2* mutation carriers, non-carriers with family history of breast cancer, and non-carriers with early-onset breast cancer

	*BRCA1* mutation carriers		*BRCA2* mutation carriers		Non-carriers with family history of breast cancer		Non-carriers with early-onset breast cancer	
	Cohort N	HR (95% CI)^1^	Cohort N	HR (95% CI)^1^	Cohort N	HR (95% CI)^1^	Cohort N	HR (95% CI)^1^
**Number of parity**								
0	44	2.13 (0.65-6.56)	61	1.12 (0.27-4.60)	162	1.49 (0.50-4.85)	274	2.13 (0.60-7.67)
1	78	1	142	1	303	1	292	1
2	79	**0.27 (0.09-0.83)**	113	0.48 (0.21-1.06)	474	1.03 (0.50-2.13)	277	0.45 (0.21-0.92)
3≤	21	**0.23 (0.05-1.00)**^2^	43	0.46 (0.13-1.33)^2^	143	0.52 (0.24-1.13)^2^	43	**4.63 (2.56-8.51)**^2^
p-trend		**<0.001**		**0.05**		**0.02**		0.50
**Age at FFTP**								
Women ≤ attained age 40								
≤23 years	20	1	27	1	90	1	82	1
24-29	54	**0.33 (0.12-0.90)**	68	1.23 (0.33-4.56)	148	2.53 (0.56-11.38)	400	1.21 (0.27-5.61)
30≤	22	**0.14 (0.03-0.66)**	38	1.14 (0.25-5.28)	91	1.38 (0.29-6.59)	126	2.68 (0.48-14.82)
p-trend		**<0.001**		0.50		0.40		0.06
Women > attained age 40								
≤23 years	12	1	46	1	132	1		
24-29	47	1.98 (0.91-4.31)	90	3.24 (1.43-7.40)	350	0.96 (0.49-1.89)		NA
30 ≤	15	1.99 (0.63-6.24)	24	1.52 (0.33-7.17)	97	0.62 (0.19-2.01)		
p-trend		**0.01**		0.60		0.10		
**Oral contraceptive use**								
Never	189	1	314	1	967	1	780	1
Ever	33	1.24 (0.45-3.40)^2^	45	0.71 (0.21-2.37)^2^	115	1.59 (0.51-5.00)^2^	106	**4.29 (2.17-9.34)**^2^

N number, HR hazard ratio, CI confidence interval, FFTP first full-term pregnancy, NA not available

^1^Adjusted for all listed variables in the table, age at first full-term pregnancy, menopausal status, age at menopause, current drinking status, and birth cohort

^2^Statistically significant heterogeneity among HRs (95% CIs) from 4 groups (p<0.001 for ≥3 parity; p=0.045 for ever oral contraceptive use)

**Table 3:** Age at menarche, age at menopause, and estrogen exposure periods for the risk of breast cancer in *BRCA 1/2* mutation carriers, non-carriers with family history of breast cancer, and non-carriers with early-onset breast cancer

	*BRCA1* mutation carriers		*BRCA2* mutation carriers		Non-carriers with family history of breast cancer		Non-carriers with early-onset breast cancer	
	Cohort N	HR (95% CI)^1^	Cohort N	HR (95% CI)^1^	Cohort N	HR (95% CI)^1^	Cohort N	HR (95% CI)^1^
**Age at menarche**								
≤ 14 years ^2^	133	1.14 (0.67-2.09)^2^	165	**3.49 (2.13-6.00)**^2^	516	**3.30 (1.73-6.34)**^2^	562	1.12 (0.57-2.33)^2^
≥ 15	89	1	193	1	564	1	324	1
**Age at menopause**								
Premenopause^2^	162	**2.27 (1.02-5.88)**^2^	222	1.13 (0.48-2.64)^2^	705	0.90 (0.27-3.02)^2^	825	0.85 (0.30-2.52)^2^
≤44 years	17	1	49	1	90	1	58	1
45-49	18	0.59 (0.28-1.62)	38	2.73 (0.70-9.98)	112	0.31 (0.08-1.18)	-	NA
50≤	18	**0.13 (0.04-0.51)**	44	0.72 (0.30-2.13)	161	**0.17 (0.05-0.61)**		
p-trend		**<0.001**		**0.05**		**0.02**		
**Estrogen exposure periods^3^**								
<25 years	125	1	162	1	337	1	802	1
25-29	48	**0.31 (0.11-0.85)**	119	0.78 (0.41-1.50)	235	**0.12 (0.03-0.37)**	71	0.41 (0.17-1.00)
30≤	42	**0.06 (0.02-0.17)**^2^	72	**0.32 (0.11-0.998)**^2^	496	**0.10 (0.04-0.25)**^2^	10	**7.69 (1.96-25.01)**^2^
		**<0.001**		**0.02**		**<0.001**		**<0.001**

N number, HR hazard ratio, CI confidence interval, NA not available

^1^Adjusted for all listed variables in the table, age at menarche, contraceptive use, number of parity, current drinking status, and birth cohort

^2^Statistically significant heterogeneity among HRs (95% CIs) from 4 groups (p=0.047 for age at menarche ≤ 14 years; p<0.001 for premenopause; p<0.001 for estrogen exposure periods≥30 years)

^3^For menopausal women, subtracting the age at menarche from the age at menopause, and for pre-menopausal females, subtracting the age at menarche from the current age

**Figure 1:**
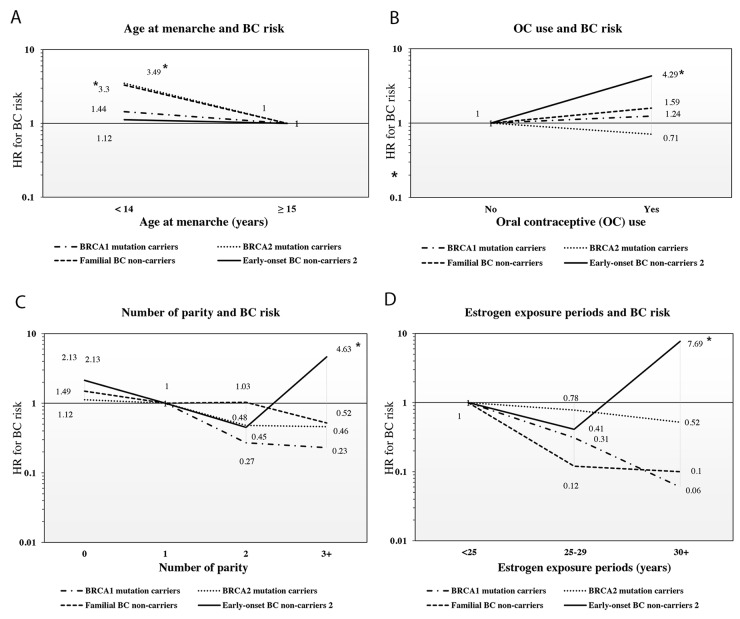
Heterogeneous variation in four HRs (95% CIs) for the risk of breast cancer in relation to age at menarche, oral contraceptive use, number of parity, and estrogen exposure periods **(A)** Age at menarche and breast cancer risk **(B)** Oral contraceptive use and breast cancer risk **(C)** Number of parity and breast cancer risk. **(D)** Estrogen exposure periods and breast cancer risk.

Earlier menarche (≤14 years old) increased the BC risk by 3.49-fold in *BRCA2* mutation carriers (95% CI=2.03–6.00) and 3.30-fold in non-carriers with FH of BC (95% CI=1.73–6.34), but was insignificantly associated with BC in *BRCA1* carriers and early-onset BC non-carriers (P-heterogeneity=0.047). Premenopausal *BRCA1* mutation carriers showed an increased risk for BC compared with *BRCA1* mutation carriers with menopausal age at <44 years old (HR=2.27; 95% CI, 1.02–5.88); however, no association was observed in other groups (p-heterogeneity <0.001). Increased age at menopause was associated with decreased risk for BC, especially in *BRCA1* mutation carriers and non-carriers with FH of BC (for menopausal women at ≥50 years old, HR=0.13, 95% CI=0.04–0.51; HR=0.17, 05% CI=0.05–0.61; P-trend <0.001 and 0.02). Longer estrogen exposure periods (LEEP, ≥30 years) were associated with higher risk for early-onset BC in non-carriers (HR=7.69; 95% CI, 1.96–25.01), but rather decreased risk for BC risk in other groups (P-heterogeneity <0.001). Figure 1 presents the association trends between age at menarche, OC use, number of parity, estrogen exposure period, and BC risk.

## DISCUSSION

We evaluated the association between reproductive factors as environmental risk modifiers and BC risk in the hereditary highly susceptible women, such as *BRCA1/2* mutation carriers and *BRCA1/2* mutation unrelated to high-risk females such as non-carriers with FH of BC and with early-onset BC.

Earlier menarche increased the risk of BC in *BRCA2* mutation carriers and non-carriers with FH of BC by threefold, but not in other two groups (HR≈1). Higher number of parity decreased BC risk by 80% in *BRCA1* mutation carriers, but rather increased the risk for early-onset BC in non-carriers by approximately fourfold. OC use was associated with increased risk for only early-onset BC in non-carriers by fourfold. LEEP and later age at menopause decreased BC risk in *BRCA1/2* mutation carriers and non-carriers with FH, whereas LEEP rather increased the risk for early-onset BC. Later age at FFTP among women aged ≤40 years was associated with decreased BC risk in *BRCA1* mutation carriers, while showing an insignificantly increased association with the other three groups.

In this study, the number of parity and BC risk was inversely associated not only in *BRCA1/2* mutation carriers but also in non-carriers with FH. However, this association was not observed in early–onset BC who were non-carriers, and rather the risk of early-onset BC was increased. Several epidemiological studies have reported that the risk of BC increases when young women give birth more than three times, which is consistent with our findings of early-onset BC [21, 22]. Excessive hormone levels from pregnancy and childbirth can promote malignant-transformed cell growth. This process can temporarily increase the risk of BC and then return to its original level with decreased hormone levels [23, 24]. More than three repetitive pregnancies and births in a very short period, especially at younger ages, may promote recurrent cumulative cell growth and thus increase the risk of BC.

Previous reports regarding the age at FFTP and BC risk among *BRCA1/2* mutation carriers were inconsistent. Some studies showed that a later age at FFTP increased the BC risk in *BRCA1/2* [15] or *BRCA2* mutation carriers [8, 9]; other studies showed no association [13, 14, 25] or decreased risk in *BRCA1* mutation carriers [8]. The inconsistency among studies might be due to the age of BC occurrence, which alters the pregnancy history, or differences in the statistical power based on the limited number of *BRCA1/2* mutation carriers included in the studies. In the general population, pregnancy increased the risk of BC in the short term, followed by a long-term risk reduction, with an effect of pregnancy on decreased BC risk among women >40 years old. This is explained by exposure to high hormonal concentrations during pregnancy, which increases the growth of preclinical tumors; however, pregnancy hormones can also reduce the susceptible cells in the breast by promoting differentiation [26]. According to this hypothesis, the analysis was stratified based on the attained age (≤40 vs. >40 years) and identified decreased risk in *BRCA1* carriers aged ≤40 years and increased risk in *BRCA1/2* carriers aged >40 years as the age at FFTP increased. Our finding that OC use increased the early-onset BC risk in non-carriers was consistent with that of a previous meta-analysis [27], suggesting that reproductive factors including endogenous and exogenous hormonal factors affected the early-onset cases more.

Results of studies associating menarche and BC risk in hereditary highly susceptible women were inconsistent. Previous Western meta-analyses [28, 29] and individual studies [12, 17] have observed a correlation between earlier menarche and higher BC risk in *BRCA1* mutation carriers, whereas we observed these associations in *BRCA2* mutations or non-carriers with FH. This result might be due to the difference in *BRCA1/2* mutation prevalence of the ethnic groups. The prevalence of *BRCA1* mutation is higher in Western populations; however, in Asian populations, *BRCA2* mutation prevalence is higher [20]. Therefore, risk modification effects of menarche may be observed only among mutation carriers with high prevalence. In non-carriers with FH, the relationship between menarche and BC risk has not been reported. However, the observed association in this study would be biologically accountable. The age at menarche has familial trait and could be regulated by other susceptible genes besides *BRCA1/2* mutations and/or gene and environment interactions [30, 31].

The alleged risk factors in the general population such as later age at menopause and longer estrogen exposure were inversely associated with high-risk women, such as *BRCA* mutation carriers and non-carriers with FH, compared with the direction in the general population because high-risk women may develop BC at an early age. The decreased association between late age at menopause (≥50 years) and BC risk or no association in *BRCA1/2* mutation carriers and non-carriers with FH were reported [10, 32], because hereditary or familial BC develops earlier than in the general population [10, 33]. In our study participants, >80% of BC patients with *BRCA1* mutation were <50 years old, and few population-based studies regarding the effects of menopause in young age groups are available, with different results compared to those in the general population.

This study had several limitations. First, the KOHBRA study population was selected based on possible hereditary traits for BC from genetic cancer clinics [34]. Effects of selection bias were still present even after minimizing them through a weighted cohort analysis to adjust for the over-representation of the affected cases [35]. Second, family members’ participation in the study would be affected by the probands’ decisions to inform and recommend genetic mutation tests because the controls were selected from the family members of BC patients who are *BRCA1* or *BRCA2* carriers. Another potential limitation was that when the duration of estrogen exposure period was calculated, parity, breastfeeding, and duration of OC use or hormonal replacement therapy were not considered [10].

Despite these limitations, the study had several strengths. Although several studies and meta-analyses existed on the associations between reproductive factors and BC risk in *BRCA1/2* mutation carriers, all studies investigated the Caucasians and no results were reported for Asian *BRCA1/2* mutation carriers until now. Indeed, to the best of our knowledge, this is the only Asian study in the literature. Although most previous studies focused on only *BRCA1/2* mutation carriers, this study targeted not only mutation carriers but also high-risk non-carriers. *BRCA1/2* mutation carriers, non-carriers with FH of BC, and non-carriers with early-onset BC were separately evaluated, and the association between the four groups were heterogeneous.

Our results indicate that reproductive factors as risk modifiers are heterogeneously associated with BC risk among these highly susceptible women, such as *BRCA1/2* mutation carriers, non-carriers with FH, and non-carriers with early-onset BC. These results are somewhat different from those in the general population. Different directions in the associations compared with the general population should be considered in the management of genetically and highly susceptible women.

## MATERIALS AND METHODS

### Study population

The KOHBRA study is a multicenter cohort study consisting of hereditary high-risk BC patients and family members of BC patients with *BRCA1/2* mutation. Hereditary high-risk BC patients were defined as BC patients with FH of breast or ovarian cancers, male BC patients, female BC patients aged ≤40 years at diagnosis, bilateral BC patients, or BC patients with other organ cancers. All patients were tested for *BRCA1/2* genetic mutations. *BRCA1/2* mutation testing was conducted using fluorescence-based conformation-sensitive gel electrophoresis, denaturing high-performance liquid chromatography, and direct sequencing in four DNA testing laboratories certified annually by the Korean Institute of Genetic Testing Evaluation. Pathogenic mutation was defined as a protein-truncating mutation and a missense mutation with confirmed association with the disease [34]. If a BC patient had a mutation, relatives aged ≥20 years were asked to participate in the study and received test for family-specific *BRCA1/2* mutation. All participants provided written informed consent and asked to complete structured questionnaires via a personal interview, including their general characteristics, past medical history, FH of malignancies, dietary and physical activities, and reproductive factors. The institutional review board of each participating center approved this study design (IRB #B-0707-047-005). Details of the study have been described fully elsewhere [34].

Of the 2,858 participants recruited from 2007 to 2014, female participants (*n*=2,684) were considered in the analysis after excluding male BC patients and male family members. After excluding six carriers with both *BRCA1* and *BRCA2* mutations, 222 *BRCA1* and 359 *BRCA2* mutation carriers were included. Of the 2,097 non-carriers of *BRCA1/2* mutation, 882 BC patients with FH of BC in the first- and second-degree relatives, 795 BC patients aged ≤40 years at diagnosis, and 201 controls were included. Appendix Figure 1 describes the details of the selection process of the participants.

#### Definitions

*BRCA1* or *BRCA2* mutation carriers were defined as women with protein-truncating mutation or a missense mutation with confirmed association with the disease within the *BRCA1* or *BRCA2* gene. Non-carriers with FH of BC were defined as women with FH of BC in first- and second-degree relatives but without *BRCA1/2* pathogenic mutation. Non-carriers with early-onset BC were defined as those who were diagnosed with BC at the age of ≤40 years but without *BRCA1/2* pathogenic mutation. Controls for non-carriers were relatives of BC patients with *BRCA1/2* mutation and found to be non-carriers after the family-specific *BRCA1/2* mutation test. Thus, all non-carrier controls were compared with non-carrier patients with FH of BC. Among them, those aged ≤40 years at recruitment were compared to non-carriers with early-onset BC (Appendix Figure 1).

As reproductive factors, we considered age at menarche (≤14 years, ≥15 years), OC use (never used, ever used), number of parity (nulliparous, 1, 2, 3≤), age at FFTP (≤23, 24-29, ≥30 years), and age at menopause (premenopause, ≤44, 45-49, ≥50 years). Estrogen exposure period was calculated by subtracting the age at menarche from the age at menopause for menopausal women, and subtracting the age at menarche from the current age for pre-menopausal women.

#### Statistical analysis

The t-test or chi-square test was used to compare the differences in the distribution of selected characteristics between those affected with BC and controls. To assess the association between the reproductive factors and BC risk, the weighted multivariate Cox proportional hazard regression model was used to retrospectively analyze the data based on the factors modifying the disease risks in carriers of highly penetrant genes to provide unbiased disease risks [35]. Weights were assigned based on the affected status, age, and *BRCA1/2* gene mutation, considering the age-specific population incidence and HR of BC for *BRCA1/2* gene mutation carriers in the Korean population [36], and the total follow-up period in affected and unaffected subjects as external rates. For non-carriers, weights were also assigned (Appendix Table 1). These weights have been adjusted for ascertainment bias caused by over-sampling of affected cases because the recruitment of participants was via the genetic clinics [35].

The HRs and 95% CIs, adjusted for family clustering and covariates, were used to determine the associations between reproductive factors and risk of BC in the four groups. To estimate the time to diagnosis, the follow-up started at birth, and subjects were censored at the age during interview and BC diagnosis for the controls and BC patients, respectively. The Cochran's Q test was used to determine the heterogeneity across the HRs (95% CIs) between the four groups [37]. All statistical analyses were conducted using the SAS software (ver. 9.1; SAS Institute, Cary, NC, USA) and Stata/SE (ver. 12.0; LP StataCorp, College Station, TX, USA).

## SUPPLEMENTARY MATERIALS FIGURES AND TABLES



## Abbreviations

BC: breast cancer
CI: confidence interval
FH: family history
FFTP: first full-term pregnancy
HR: hazard ratio
LEEP: longer estrogen exposure periods
OC: oral contraceptive
