# Supplementary material for: Reproductive factors as risk modifiers of breast cancer in BRCA mutation carriers and high-risk non-carriers
Source: Oncotarget. 2017 Oct 31;8(60):102110–8. doi: 10.18632/oncotarget.22193 (PMC5731939; doi:10.18632/oncotarget.22193)
Supplement: Supplementary file 1 [file oncotarget-08-102110-s001.pdf]

Reproductive factors as risk modifiers of breast cancer in *BRCA* mutation carriers and high-risk non-carriers

SUPPLEMENTARY MATERIALS

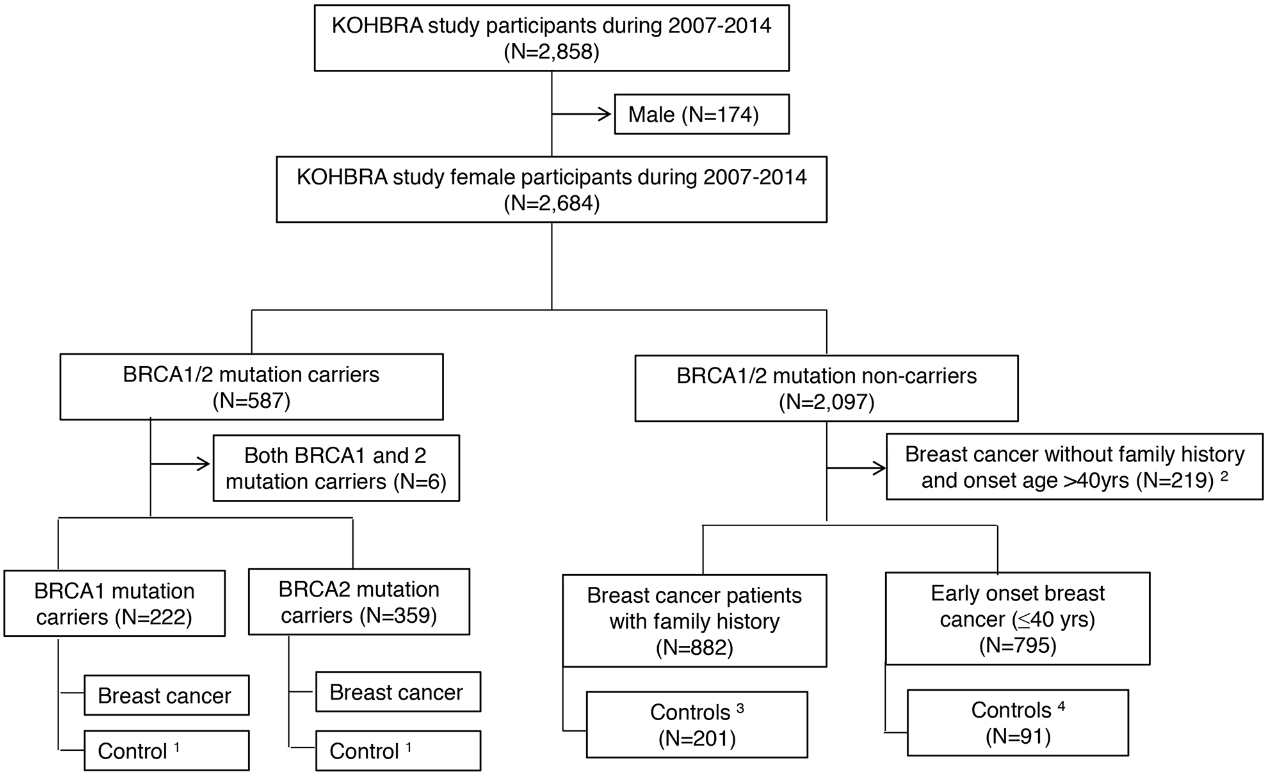

Appendix Figure 1: Flow chart of study participants selection.

[1]Controls were the BRCA1 or 2 carriers from the 1st and 2nd degree relatives of breast cancer patients with BRCA1/2 mutation

[2]Include bilateral breast cancer patients or breast cancer patients with other organ cancer diagnosed >40yrs and without family history of breast cancer

[3,4]Controls were relatives of breast cancer patients with BRCA1/2 mutation and found to be non-carriers after family-specific BRCA1/2 mutation test

[4]Among 201 controls who had family history of breast cancer, 91 controls aged ≤ 40 yrs were compared with early onset breast cancer.

Appendix Table 1: Incidence rate of breast cancer in Korea and assigned weights for *BRCA1*/2 mutation carriers and high-risk non-carriers.

Age group	Incidence rate of breast cancer[2] (/100,000)	Weights for *BRCA1* mutation carriers		Weights for *BRCA1* mutation carriers		Weights for *BRCA* mutation non-carriers [1]	
		Case	Control	Case	Control	Case	Control
18-24	1.9	0.0734	1.9266	0.1190	1.4111	0.0145	1.8916
25-29	8.3	0.1028	2.5700			0.0054	7.7362
30-34	26.7	0.0873	5.1985	0.1034	3.8276	0.0057	16.9508
35-39	61.0	0.1082	11.7014	0.1315	5.5003	0.0067	22.2316
40-44	100.8	0.2311	3.2107	0.2085	2.7589	0.0164	10.8772
45-49	140.7	0.2975	3.6343	0.1892	3.8378	0.0117	18.1495
50-54	128.4	0.2168	3.1539	0.2186	2.2022	0.0119	6.6122
55-59	108.9	1.1197	0.9402	0.0771	4.5379	0.0110	5.4798
60-64	99.9	0.2706	1.3647	0.1239	2.7523	0.0073	3.5692
65-69	81.6	0.0214	1.3262	0.8985	1.2369	0.0049	6.4728
70-	63.8					0.0025	2.4963

[1]Breast cancer patients with family history of breast cancer, early age onset (<40), past and current ovarian cancer, and past and current BRCA-mutation-related cancers such as pancreatic, laryngeal, and colon cancer
[2]In the year 2010
